# The Role of Affective Reactivity Induced by Cigarette Packaging including Graphic Warning Labels: The CASA Study

**DOI:** 10.1136/tobaccocontrol-2021-056650

**Published:** 2021-09-12

**Authors:** Matthew D. Stone, David R. Strong, Claudiu V. Dimofte, Elizabeth Brighton, Jesica Oratowski, Tingyi Yang, Manar Alkuzweny, Atean Asslani, Katherine Velasco, Michael Skipworth, Noe C. Crespo, Samantha Hurst, Eric Leas, Kim Pulvers, John P. Pierce

**Affiliations:** 1 Herbert Wertheim School of Public Health and Human Longevity Science, University of California, San Diego; 2 School of Public Health, San Diego State University, San Diego; 3 Moores UC San Diego Cancer Center, University of California, San Diego; 4 Department of Marketing, San Diego State University; 5 Department of Psychology, California State University San Marcos

## Abstract

**Objective:**

To identify whether three types of cigarette pack designs, including three versions of Graphic Warning Label (GWL) plain packs, one GWL absent and branding absent pack (Blank) and the smoker’s own GWL absent and branding present pack (US), elicit different valence, type and levels of affect.

**Design:**

US daily smokers (n=324) were asked to handle each of the five pack types and “Think Aloud” their reactions. To avoid a muted familiarity response, exposure to their own US pack followed exposure to at least one GWL plain pack. Reactions were scored on a reactivity scale (−3 to +3) and the text was coded for speech polarity (−1 to +1) and emotive word frequency.

**Results:**

Reactivity scores had excellent inter-rater reliability (agreement≥86%; ICC≥.89) and were correlated with speech polarity (*rho*’s=.21-.37, *p*-values<.001). When considering their US pack, approximately two-thirds of smokers had a low (31.5%) to medium (34.6%) positive response (reactivity=1.29; polarity=0.14) with expressed feelings of joy and trust. Blank packaging prompted a largely (65.4%) neutral response (reactivity=0.03; polarity=0.00). The gangrenous foot GWL provoked mostly medium (46.9%) to high (48.1%) negative responses (reactivity=−2.44; polarity=−0.20), followed by neonatal baby (reactivity=−1.85; polarity = −0.10) and throat cancer (reactivity=−1.76; polarity=−0.08) warnings. GWLs varied in their elicitation of disgust, anger, fear, and sadness.

**Conclusion:**

Initial reactions to GWL packs, a blank pack, and smokers’ current US pack reflected negative, neutral, and positive affect, respectively. Different versions of the GWL pack elicited different levels and types of immediate negative affect.

## Introduction

Cigarette packaging offers a point-of-use marketing opportunity to influence both a smoker’s behavior and the perceptions of observers, particularly young people.^1–3^ Branded marketing on cigarette packages is associated with positive affect that supports the decision to smoke another cigarette.^4–6^ Completely removing industry marketing from the packaging may not be sufficient to counteract positive affect^7^ and inhibit incentive salience attribution.^8, 9^ Graphic warning labels (GWLs) of the health consequences of smoking aim to introduce negative affect with the goal of having the smoker reconsider the decision to smoke. As of January 2021, 127 countries have mandated GWLs on cigarette packaging,^10^ and 17 countries have mandated plain packaging pioneered by Australia,^11^ which removes industry branding and adds GWLs on 75% of the pack.^12, 13^ The United States is the only high income country that has not yet mandated GWLs on cigarette packs.

Multiple studies have shown that GWL packaging is associated with negative affect.^14–19^ Yet, the measurement of affective response to emotion-evocative stimuli is complicated, with self-report measures requiring recalled responses to the packaging without the presence of the stimulus.^20^ The GWL literature mainly uses brief self-report paper and pencil measures of affect, resulting in a simple quantitative scale. Such a measure may be complemented by additional research using observational methods that add rich context.^21^

The type of affect that cigarette packaging might induce is thought to be a minor “emotional episode”.^22^ Viewing a GWL package may elicit a minor valenced reaction that would not be strong enough to elicit any major physiological activation (such as fight or flight response), but is enough to have individuals think about their decision to smoke.^23^ People are known to use emotive words to express the affect they feel when reacting to such an episode and the act of describing their response often helps them regulate their emotions.^24^ A “think aloud” technique^25^ asks smokers to explore cigarettes packaging and express their thoughts and feelings as they undertake the exploration.^26^ This approach elicits verbalized spontaneous thoughts^27^ about the pack presented, that is often influenced by cognitions and emotions from previous experiences with the product.^28^ This approach is most fruitful when different packaging options are compared, particularly when a very familiar pack is explored after they have been challenged with a pack featuring negative emotive stimuli.^29^ This observational methodology when paired with multi-method measurement^30^ of responses can capture immediate reactivity, opposed to paper-pencil measures^31^ which may promote evaluative reactions.

An examination of real-world emotional responses to various cigarette packaging designs is needed to help inform how GWLs may influence smoking cognitions and behavior, should they be introduced in the US in July 2022.^32^ This paper aims to establish and validate an immediate reactivity measure and identify whether the three types of pack designs elicit distinct affective valence. In this study we use a structured pack handling task with a “think-aloud” cognitive interview to identify the individual variability in how US cigarette smokers react to 5 different cigarette packaging options: three plain packs with different GWLs, one blank pack devoid of branding and GWLs, and their usual US cigarette packs (branded without GWLs). We hypothesize reactions to US pack will fall along a range of positive affect, the variability for the blank pack will be in the neutral range, and reactions to all GWL packs will fall along a range of negative affect. All participants in this study were enrolled in a randomized trial where they received 3 months real world experience with their cigarettes repackaged into plain packs with GWLs, blank packs, or maintained their usual US pack. We expect that the cognitive and behavioral responses in the trial will be determined by the immediate reactivity that the participants had to each of the study packs.

## Methods

### Study Population:

This study uses cross-sectional data collected during the initial in-person visit (V1) for the CASA randomized trial of the effects cigarette packaging on smoking cognitions and behavior.^33^ Volunteer daily smokers, aged 21–65 years from San Diego County, California, were enrolled using community advertising. All participants signed an informed consent (overseen by Institutional Review Boards at UC San Diego and Cal State San Marcos), completed questionnaires, and followed a protocol to think aloud their reactions as they explored study cigarette packaging.

### Pack Handling Task:

Participants were handed one pack at a time and asked to verbalize what thoughts came to their mind as they explored each side of each pack. For each pack, verbalizations were timed, recorded, and transcribed. There were 5 study packs (eFigure 1) each labelled with the participant’s brand and variant: three GWL plain packs; one blank pack (devoid of marketing with GWLs absent); and their current US pack (branding present but GWL absent). In a pre-test^33^, we selected 3 of 8 GWL-plain packs licensed from the Commonwealth of Australia using negative affect scores from the Positive and Negative Affect Scale.^34^ We were concerned that familiarity with their own pack might lead to only cursory attention if it was presented first in the pack handling task. Accordingly, we required at least one GWL pack to be presented as the first pack which focused the individual’s attention on the packaging elements that were being changed. GWL packs were thus randomized to the 1st, 3rd, or 5th presentation and the blank pack and US pack to the 2nd or 4th presentation.

### Coding Reactivity to Study Packaging:

Using a multi-method qualitative approach,^30, 35^ two coders in consort with an anthropologist (SH) developed a coding manual^36^ for a 7-point affect scale (high, medium, low for both negative and positive reactivity as well as a central neutral category; Table 1) using a training set of 30 transcriptions. Four additional coders were trained using this set until group concordance (±1) was reached on 80% of transcriptions. In total, six coders used the coding manual to independently rate each transcription for each pack The coders met weekly to discuss their scores and resolve instances of coding discordance. High reactivity was indicated by use of highly emotional words or amplified moderately emotional words that suggested a somewhat visceral reaction to the packaging. If moderately emotional words or highly emotional words were used and de-amplified (e.g., “*somewhat* disgusting”) or emotional statements accompanied by qualifications (e.g., “that’s disgusting but *it would not stop me from smoking*”), that indicated medium reactivity. A low level was a mild reaction followed by a rationalization. Neutral reactivity was when no emotional or reactive language was uttered. For each pack, reactivity scores were averaged, and categorical reactivity scores generated by rounding mean scores to their nearest integer.

### Natural Language Processing of Initial Reactivity:

Using R version 4.0.3 with the ‘SentimentR’ package,^37^ we conducted natural language processing of the transcribed speech from the pack handling task to quantify the number of words uttered and polarity of word choice. Using the Jockers–Rinker sentiment lexicon of 11,710 polarized words,^38^ sentences were classified according to their overall polarity (e.g., the degree to which the speech and its linguistic modifiers had a positive, neutral, or negative valence; eTable 1). To account for extreme negative words occurring more commonly in natural language,^39^ polarity scores were scaled from −1 to +1 using a general rescaling function.^37^ Linguistic modifiers were accounted for by examining the four words following, and two words preceding, each polarized word and tagged as one of the following: neutral, negators (flip the ± polarity sign of a word, e.g., “I do *not* like it”), amplifiers or de-amplifiers (increase or decrease the impact of a word by multiplying polarity scores using standard preset weights.^38^, e.g., “I *really* like it. I *hardly* like it”), or conjunctions (overrule previous clauses, e.g. “I like it *but it’s not worth it*”). The sentiment lexicon was augmented to neutralize polarized words that had different connotations in our study (e.g., baby, child, surgeon). Sentence-level polarity scores were averaged to generate composite polarity scores per participant per pack. The prototypical emotions of fear, disgust, anger, sadness, anticipation, trust, joy, and surprise^40^ were explored using ‘SentimentR’s’ emotion function and the NRC Hashtag Emotion Lexicon look-up of 8265 emotion terms.^41, 42^ The rate of emotion expressed was evaluated as the number emotional words uttered relative to the total number of words spoken, with scores ranging between 0 (no emotional utterances) and 1 (all emotional utterances).

### Study Covariates:

Sociodemographics (age, sex, race/ethnicity, and educational attainment),^33^ tobacco use (daily use frequency and primary brand smoked),^33^ the Fagerström Test of Nicotine Dependence scale,^43^ brand loyalty,^4^ and health anxiety^44^ were measured covariates. We assessed brand appeal using a 6-point Likert scale (‘The design on the brand of cigarettes I currently smoke is…Stylish, Fashionable, Cool, High quality, Attractive, Appealing’; α = .92).^45, 46^

## Statistical Analysis

Inter-rater reliability of the coded reactivity scores across the five pack conditions was evaluated in two ways:^47^ a) by computing the percentage agreement across the scores while allowing for a tolerance of 1 in ratings, and b) by modeling the intraclass correlation coefficient (ICC) among the raters. With the goal of constructing composite scores, a two-way random effects (i.e., participants within pack type) ICC model was used^47^ with raters’ scores evaluated for consistency.^48^ To examine patterns in highest levels of reactivity, quintile cut points were calculated. To examine differences in the time to explore packs, total words uttered, polarity of word choice and verbalized reactivity expressed, we conducted Kruskal-Wallis tests and post-hoc examination of pairwise comparisons using Dunn’s tests. Spearman Rho correlation coefficients were used to evaluate construct validity between reactivity scores and word polarity. To explore differences in emotion expressed during pack handling, we plotted the average rates of emotional utterances using a radar chart.^49^ To explore the associations between sample characteristics and reactivity scores, we fit an intercept only conditional mixed-effects model with bootstrapped confidence intervals using the “Lme4” package. Reactivity scores were the outcome of interest, with package viewing order, age, gender, race/ethnicity, education, health anxiety, nicotine dependence, brand appeal, brand loyalty, and brand smoked included as fixed effects. All two-way interactions between pack condition and covariates were examined using the “LmerTest” package and significant terms (*p*<.05) retained using an omnibus F-test. Estimated marginal means were computed from model terms using the “effects” package and then plotted.

## Results

We obtained quality transcriptions from 324 of the 357 participants of the CASA trial (91%). The average age in our sample was 39.3 years (SD=11.8), 47% were female, 68% were non-Hispanic White, with 41% having received a college degree. (eTable 2) Participants had low generalized health anxiety scores (Mean=1.1, SD=.09) and smoked 11.6 (SD=5.9) cigarettes/day (Mean=11.6, SD=5.9), with moderate levels of nicotine dependence (Mean=3.8, SD=2.3). The majority (77%) reported loyalty to a cigarette brand (Marlboro=43%; Camel=26%; American Spirit=18%) as well as high levels of appeal towards their brand’s packaging (Mean=3.7, SD=1.2).

### Assessing the ‘Think-Aloud’ Pack Handling Task:

Quality data on pack handling time was limited to 234 participants (72%). Average pack handling times were: Own pack (59.4 seconds), Blank pack ( 47.0 seconds) and GWL plain pack (80.2 seconds; Table 2). The average number of words in the “think aloud” were: Own pack (97 words), Blank pack (69 words) GWL plain pack (110 words). Inter-rater reliability (±1 tolerance) for reactivity scores of the six coders ranged from a low of 86.1 for their own US pack to a high of 97.8 for the foot gangrene GWL pack. The ICCs were also very high for all five reactivity scores (range: 0.89 to 0.95). Less than 2% of participants commented that they had previous experience with GWLs packs.

Three quarters of reactivity scores for participants’ own packs were positive (high positive =9.2%; medium positive=34.6%; low positive=31.5%), for an overall mean reactivity score of 1.29 (95%CI=1.25, 1.34). Reactivity scores for the blank pack were mainly neutral (low positive=15%, neutral 65.4%, low negative 15%) for an overall mean score of 0.03 (95%CI=0.00, 0.07). Reactivity scores for each of the 3 GWL plain packs were heavily negative: Throat cancer: high negative=8.6%. medium negative=64.5%, low negative=24.1% for an overall mean reactivity score of −1.76 (95%CI=−1.79, −1.73); Neonatal Baby: high negative=11.7%. medium negative=62.7%, low negative=21.3%, for an overall mean reactivity score of −1.85 (95%CI=−1.89,−1.82); Foot Gangrene: high negative=48.1%. medium negative=46.9%, low negative=4.6%, for an overall mean reactivity score of −2.44 (95%CI=−2.47,−2.41). When we examined quintiles of reactivity across the US and GWL packs, we found that 66.7% were highly reactive (top quintile) to at least one pack while 88.9% were moderately reactive (top two quintiles) to at least one pack. Only 8.3% of subjects were highly reactive to three or more packs.

The language processing analysis of the polarity of the words used in the “think aloud” task showed a pattern similar to the coded reactivity scores across design conditions: US pack, polarity mean=0.14 [95%CI=0.13, 0.15]; Throat cancer polarity mean =−0.08 [95%CI=−0.08, −0.07]; Neonatal baby, polarity mean=−0.10 [95%CI=−0.11, −0.09); Foot Gangrene, polarity mean= −0.20 [95%CI=−0.21, −0.19]). For each pack condition, polarity scores were correlated with mean reactivity scores (Spearman Rho’s range: 0.30–0.38, *p-*values <.001). Overall, both reactivity scores (*p*-values <.001) and polarity scores (*p*-values <.001) were significantly different across each packaging design condition.

**Figure 1.**
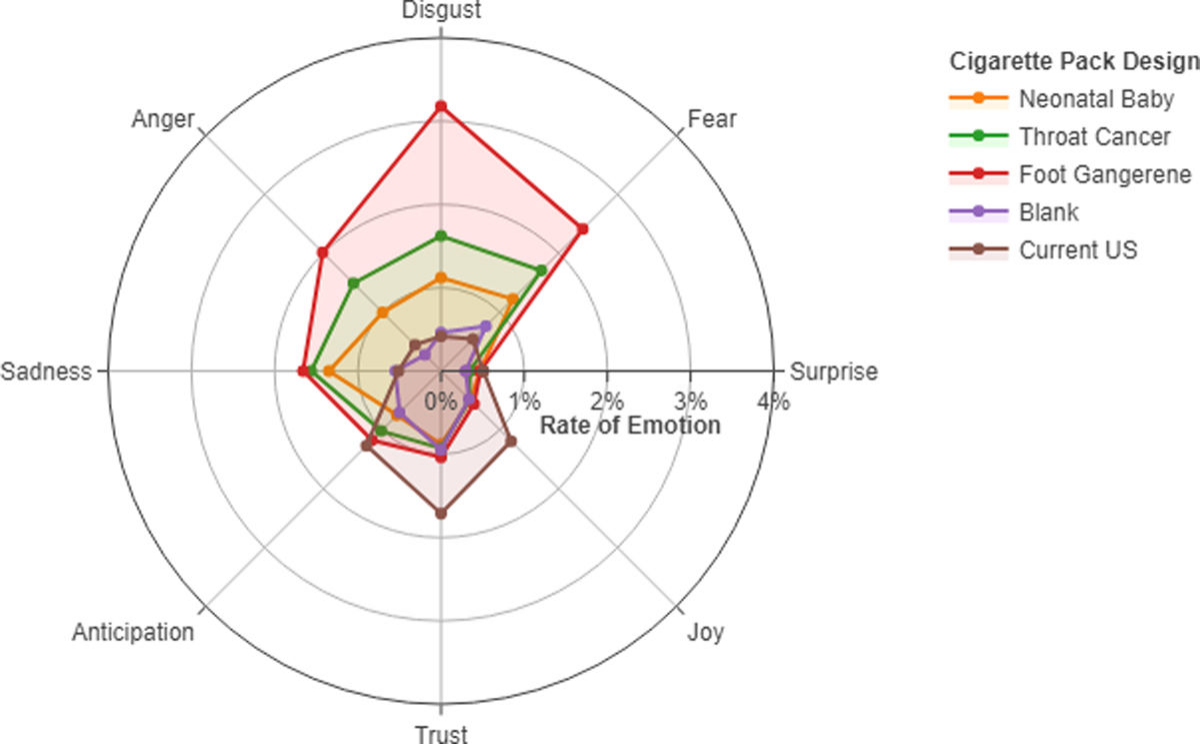
Average rate of emotive words spoken during pack exposure period (n=324). A semantic analysis of transcribed speech that was text mined for emotive utterances using an emotion word lexicon and computing the rate of emotive words expressed per sentence between 0% (no emotional utterances) and 100% (all emotional utterances).

The frequency of prototypical emotions expressed in the “think aloud” is presented in the radar chart (Figure 1). The foot gangrene pack elicited more emotions characterized as disgust, fear and, to a lesser extent, anger. A similar distribution of expressed emotions was seen in response to the throat cancer GWL pack, although at a lower frequency. The primary emotion elicited by the Neonatal Baby GWL pack was sadness. The two main emotions elicited by their own pack were trust and joy.

### Predicting Reactivity to Cigarette Packaging Designs:

**Figure 2.**
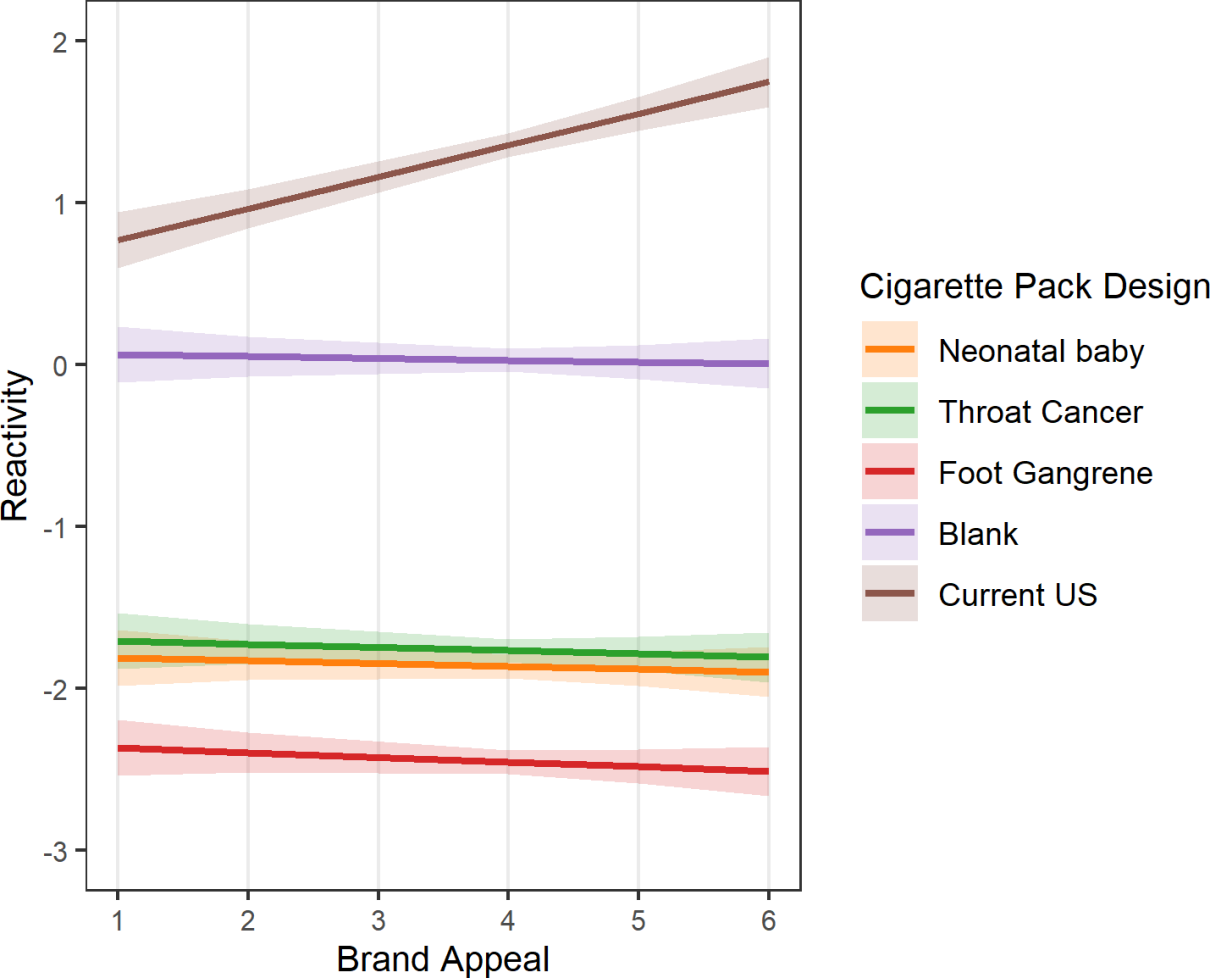
Relationship between level of brand appeal and reactivity to five cigarette pack designs (N=324). Estimated marginal means and 95% confidence interventions extracted from intercept only conditional mixed-effects model predicting reactivity to cigarette packaging design with age, race/ethnicity, education, health anxiety, nicotine dependence, brand loyalty, and brand smoked included as fixed main effects and package viewing order, gender, and brand appeal as interaction effects.

The model of reactivity scores (Table 3) had main effects for pack type (F[4,1589]=59.76, *p*<.001), and health anxiety (F[1,1589]=12.14, *p*<.001), and interactions between pack type by viewing order (F[4,1589]=4.68, *p*<.001), gender (F[4,1589]=8.09, *p*<.001), and brand appeal (F[4,1589]=10.54, *p*<.001). Compared to the blank pack, reactivity scores for their US pack were significantly more positive for each increasing level of brand appeal (β=0.21 [95%CI=0.13, 0.29], *p* <.001). Those with greater brand appeal ratings expressed more positive reactivity scores for their own packs (The 75^th^ percentile level of brand appeal had a reactivity score of 1.45 [95%CI=1.37, 1.54] which was much higher than the 25^th^ percentile level with a score of 1.16 [95%CI=1.08, 1.24]; Figure 2). No relationship was observed between ratings of brand appeal and reactivity scores for GWL or Blank packaging. More positive reactivity scores for the US pack were observed when the pack was viewed later in the pack handling task (4^th^ position=1.43 [95%CI=1.33, 1.53]) compared to when it was viewed earlier in the task (2^nd^ position=1.16 [95%CI=1.06, 1.26]).

## Discussion:

US daily smokers, with minimal previous exposure to GWLs, demonstrated consistent negative reactions when they were exposed to the GWLs used as part of plain packaging licensed from the Commonwealth of Australia. While reactivity to GWL packaging was negative across the board, the level of reactivity appeared to align with the negative emotional response found in prior work.^33, 50–53^ Conversely, smokers’ current branded US cigarette pack was associated with positive reactivity which was higher when their branded pack occurred after exposure to two different GWL packs in the study’s pack handling protocol. This finding supports previous research that found current cigarette packaging in the US to be associated with positive affect for smokers, which may promote more regular smoking behavior.^4^ Blank packs, devoid of all marketing, drew a neutral response. Thus, this study has established that these three packaging conditions (GWL plain pack, Blank pack, US pack) elicit markedly different initial participant reactions to the cigarette packaging.

A major objective of Australia’s GWL health consequences messaging was to induce thoughts (e.g., “I cannot bear to think of that happening to me”) that might promote future quitting behavior.^54^ Notably, this study found a significant negative affect experienced by US smokers in response to the GWL plain packs, most markedly with the foot gangrene image. Both the images of the neonatal baby and throat cancer were associated with negative affect where the emotions appeared to be a mix of fear, disgust, anger, and sadness – which appear consistent with the goal of this health consequences messaging.^54^ However, the foot gangrene image was associated with much stronger negative emotions that were more likely to be characterized as visceral. The emotions expressed appeared to be disgust, fear, and anger much more than sadness. In future work, we aim to explore the transcribed text for the directionality of anger, which could be directed at the tobacco industry,^55^ governmental regulations,^56^ or somewhere else. One of the strengths of this qualitative methodology is that it facilitates such further detailed analyses. In the randomized trial, we aim to use ecological momentary assessment to test whether the high initial reactivity to the GWL packs images is associated with increased cognitions when participants reach for a cigarette.^57^ With twice daily measurement, we will be able to assess whether and how this reactivity is associated with avoidance and/or pack hiding behavior,^58^ which may^59^ or may not^60^ be related to cessation behaviors. The detailed and frequent measurement of both cognitions and behavior in the randomized trial will be a major advance on most of the studies completed to date.^23,59^

GWL plain packs may disrupt the incentive salience attributed to the cigarette packaging via the removal of industry marketing and inclusion of visceral imagery and aversive design characteristics (e.g., fonts and colors). Cue-learning models suggest that appealing design features on packaging capture attention, generate positive affective reactions, and motivate behavior that may facilitate a desire to smoke.^61, 62^ We found that the more brand appeal smokers reported for their own US marketed pack (e.g., cool, stylish, etc.), the more positive their reaction was when asked to express their thoughts and feelings about it. When appealing marketing cues are affixed to tobacco products and perceived immediately prior to use, the cues themselves can acquire similar motivational significance and evoke a desire to smoke.^8, 63, 64^ Yet, levels of brand appeal did not influence the reactivity to the GWL plain packs, despite the packs being matched to the smoker’s cigarette preference and clearly labeled with brand and variant name. Thus, plain GWL packaging may have the intended effect of inhibiting incentive salience attribution by quelling the appeal of the product, an effect consistent with prior research suggesting that plain GWL packaging impedes the product’s ability to generate appeal.^65–67^ Nevertheless, reactivity to the blank pack did not vary by levels of brand appeal, indicating that the appeal of the product may be suppressed by simply removing tobacco industry marketing.

There are a number of factors that limit the generalizability of these findings: a) the study recruited volunteer smokers and the sample was not representative of the US population or smokers in other countries; b) under-representation of minorities in the study also resulted in a lower proportion of menthol smokers; c) all participants were from California which has stronger social norms against smoking than the rest of the US.^68^ Other limitations included the loss of < 10% (n=33) of the ‘think aloud’ data due to a computer hardware failure at our storage facility that was unrelated to the trial, indicating that the data are most likely missing-at-random.^69^ The GWL packaging proposed for use in the US is not on the plain packaging used in this study, but a hybrid packaging condition that includes reduced industry marketing with smaller graphic warning labels, a design quite common in many countries.^12^ We would expect that such hybrid packaging would be associated with a lower level of initial reactivity to the GWLs than was observed in this assessment.

Despite limitations, the study had numerous strengths. It allowed smokers to openly express their thoughts and feelings about GWL packaging, thus resulting in more rich emotive details than structuring their response through a questionnaire. All study packs were matched to the participants’ preferred cigarette brand and variant in an effort to maintain cigarette expectancies and isolate the effects of the reactivity. We used observational measurement of reactions to the various pack designs with high-quality coding, which yielded a full range of valenced reactivity and was concurrently valid with the polarity of speech as identified by natural language processing. We used an exposure to GWL plain packs prior to assessing reactivity to US packs which likely focused the participant’s thinking on what they liked about their current pack, resulting in more reliable reactions.

## Conclusion

GWLs are an integral part of the recommended suite of tobacco control strategies for governments to reduce the health costs associated with disease caused by cigarettes.^13^ Yet, to date litigation by the tobacco industry has blocked implementation in the US by arguing that GWLs are too aversive and are aimed at forcing smokers to quit.^70^ In this study, we have demonstrated that US smokers do react with a range of negative emotive reactions to GWL packs, contrasting with the appeal of their regular branded pack. Future studies are needed to demonstrate whether GWL packaging achieves FDA’s goal of encouraging smokers to think about the health consequences of using these products.

## Supplementary Material

Supp1

Supp2

## Figures and Tables

**Table 1.** Coding System for Reactivity to Each Study Pack

Rating	Participant Reactions and Descriptions of Study Packs Include:
**High Negative** *Score: −3*	Highly emotional words or amplified moderately emotional words that are negatively valanced to describe pack aversion. Visceral reaction and repeated exclamations of aversion; might repeat emotional words. Language that indicates they do not want to handle the pack.
**Medium Negative** *Score: −2*	Moderately emotional words or de-amplified highly emotional words that are negatively valanced to describe pack aversion. No visceral reaction and a lower emotional response than high aversion. Strong initial negative reaction followed by rationalization (e.g., pack design would not modify behavior).
**Low Negative** *Score: −1*	Moderately emotional words that are negatively valanced followed by detracting statements or de-amplifiers that overrule the response. No visceral reaction or high/moderate negative emotional response. Mild reaction or acknowledgement of pack aversion followed by rationalization (e.g., pack design would not modify smoking behavior).
**Neutral** *Score: 0*	No emotional words to describe pack. No or little reaction to the pack and/or appear to be unaffected by the pack. Text on the pack may be read without saying how it makes them feel.
**Low Positive** *Score: +1*	Moderately emotional words that are positively valanced followed by detracting statements or de-amplifiers that overrule the response. No visceral reaction or high/moderate positive emotional response. Mild reaction or acknowledgement of pack appeal followed by rationalization (e.g., pack design would not modify smoking behavior).
**Medium Positive** *Score: +2*	Moderately emotional words or de-amplified highly emotional words that are positively valanced to describe pack appeal. No visceral reaction and a lower emotional response than high appeal. Strong initial positive reaction followed by rationalization (e.g., acknowledgement of the health consequences of smoking).
**High Positive** *Score: +3*	Highly emotional words or amplified moderately emotional words that are positively valanced to describe pack appeal. Visceral reaction and exclamations of appeal; might repeat emotional words. Language that indicates a desire to smoke a cigarette.

**Table 2.** Examination of Verbalized Response to Study Pack Designs during Initial Exposure using Coded Reactivity and Natural Language Processing (n=324)

	Cigarette Packaging Design					
Characteristic	Current US	Blank	Throat Cancer	Neonatal Baby	Foot Gangrene	*P*-value^7^

**Seconds Held** ^1,2^	59.4 (56.1, 62.7)	47.0 (44.5, 49.6)	78.5 (74.7, 82.3)	78.5 (74.3, 82.8)	83.7 (79.4, 88.2)	<.001
**Language Processing** ^1^						
Words uttered	96.6 (91.9, 101.4)	69.2 (65.6, 72.8)	109.7 (104.7, 114.7)	110.0 (103.9, 116.2)	104.8 (99.9, 109.6)	<.001

Speech polarity	0.14 (0.13, 0.15)	0.00 (−0.01,0.01)	−0.08 (−0.08, −0.07)	−0.10 (−0.11, −0.09)	−0.20 (−0.21, −0.19)	<.001
**Coded Reactivity**						
Mean score	1.29 (1.25, 1.34)	0.03 (0.00, 0.07)	−1.76 (−1.79, −1.73)	−1.85 (−1.89, −1.82)	−2.44 (−2.47, −2.41)	<.001
Categorical score^3,4^						
*High negative*	0 (0.0%)	0 (0.0%)	28 (8.6%)	38 (11.7%)	156 (48.1%)	

*Medium negative*	0 (0.0%)	2 (0.6%)	209 (64.5%)	203 (62.7%)	152 (46.9%)	

*Low negative*	1 (0.3%)	50 (15.4%)	78 (24.1%)	69 (21.3%)	15 (4.6%)	

*Neutral*	79 (24.4%)	212 (65.4%)	9 (2.8%)	14 (4.3%)	1 (0.3%)	

*Low positive*	102 (31.5%)	55 (17.0%)	0 (0.0%)	0 (0.0%)	0 (0.0%)	

*Medium positive*	112 (34.6%)	5 (1.5%)	0 (0.0%)	0 (0.0%)	0 (0.0%)	

*High positive*	30 (9.2%)	0 (0.0%)	0 (0.0%)	0 (0.0%)	0 (0.0%)	
Inter-rater reliability^5,6^						
*ICC*	0.95 (0.94, 0.96)	0.93 (0.92, 0.95)	0.90 (0.89, 0.92)	0.93 (0.91,0.94)	0.89 (0.87, 0.91)	

*Agreement ± 1*	86.1%	92.9%	93.8%	94.4%	97.8%	

1 Statistics presented: mean (95% confidence interval) on a 7-point affect scale (−3 to +3)

2 A subsample of cases were available for timing of the pack handling task (n=234).

3 Statistics presented: n (%)

4 Rounded rater coded reactivity score

5 Intraclass Correlation Coefficient (95% confidence interval) for coded reactivity score across six independent raters

6 Interrater agreement allowing for a tolerance of 1 in ratings.

7 Statistical tests performed: Kruskal-Wallis Test.

**Table 3.** Associations between Sample Characteristics and Reactivity to Cigarette Packaging Designs (n=324)

	Reactivity^			
	Main Effects Model		Interaction Model	
Regressor	β (95%CI)	*P*-value	β (95%CI)	*P*-value
**Main Effects**				
Viewing order	0.01 (−0.02, −0.03)	.56	−0.02 (−0.09, −0.05)	.53
Pack				
*Blank*	*Ref*		*Ref*	
*Current US*	1.26 ( 1.15, −1.36)	<.001	−0.13 (−0.59, −0.34)	.57
*Throat Cancer*	−1.79 (−1.89, −1.69)	<.001	−1.74 (−2.16, −1.35)	<.001
*Neonatal Baby*	−1.89 (−2.00, −1.79)	<.001	−1.90 (−2.32, −1.47)	<.001
*Foot Gangrene*	−2.48 (−2.58, −2.38)	<.001	−2.24 (−2.69, −1.84)	<.001
Age (per 10 years)	0.01 (−0.02, −0.04)	.56	0.01 (−0.02, −0.04)	.61
Gender				
*Male*	*Ref*		*Ref*	
*Female*	−0.11 (−0.18, −0.04)	<.001	−0.05 (−0.18, −0.09)	.52
Race/Ethnicity				
*Non-Hispanic White*	*Ref*		*Ref*	
*Hispanic*	−0.05 (−0.15, −0.06)	.36	−0.04 (−0.14, −0.06)	.43
*Other Non-Hispanic*	0.04 (−0.05, −0.12)	.38	0.04 (−0.05, −0.12)	.36
Education				
*College or advanced degree*	*Ref*		*Ref*	
*Some college*	0.03 (−0.05, −0.09)	.47	0.02 (−0.05, −0.09)	.54
*High school or less*	−0.08 (−0.18, −0.03)	.17	−0.09 (−0.20, −0.02)	.10
Health anxiety	−0.07 (−0.11, −0.03)	<.001	−0.07 (−0.10, −0.03)	<.001
Nicotine dependence	−0.00 (−0.02, −0.01)	.53	−0.01 (−0.02, −0.01)	.51
Brand appeal	0.03 ( 0.00, −0.05)	.08	−0.01 (−0.07, −0.04)	.71
Brand smoked				
*Marlboro*	*Ref*		*Ref*	
*American Spirit*	−0.04 (−0.14, −0.05)	.37	−0.04 (−0.14, −0.05)	.35
*Camel*	0.05 (−0.03, −0.14)	.19	0.05 (−0.03, −0.13)	.22
*Other*	0.01 (−0.09, −0.12)	.79	0.01 (−0.09, −0.12)	.82
Brand loyalty				
*No*	*Ref*		*Ref*	
*Yes*	0.03 (−0.05, −0.12)	.42	0.03 (−0.05, −0.11)	.43

**Interactions**				
Pack × Viewing order				
*Blank*	--		*Ref*	
*Current US*	--		0.16 ( 0.06, −0.26)	.002
*Throat Cancer*	--		0.02 (−0.07, −0.10)	.68
*Neonatal Baby*	--		0.04 (−0.04, −0.13)	.30
*Foot Gangrene*			−0.01 (−0.09, −0.07)	.77
Pack × Gender *(Ref* = Male)				
*Blank*	--		*Ref*	
*Current US*	--		0.28 ( 0.08, −0.47)	.007
*Throat Cancer*	--		−0.13 (−0.32, −0.07)	.22
*Neonatal Baby*	--		−0.18 (−0.38, −0.02)	.08
*Foot Gangrene*	--		−0.24 (−0.44, −0.05)	.019
Pack × Brand appeal				
*Blank*	--		*Ref*	
*Current US*	--		0.21 ( 0.13, −0.29)	<.001
*Throat Cancer*	--		−0.01 (−0.10, −0.07)	.83
*Neonatal Baby*	--		−0.01 (−0.09, −0.08)	.87
*Foot Gangrene*	--		−0.02 (−0.10, −0.07)	.67

*Note*. From separate intercept only conditional mixed effects models with bootstrapped 95% confidence intervals (n=1000) predicting reactivity to cigarette packaging design.

^ Measured on 7-point affect scale (−3 to +3: high, medium, low for both negative and positive reactivity as well as a central neutral category)

## What this paper adds:

What is already known on this subject?

- Graphic warning labels (GWLs) on cigarette packaging have been implemented in 120+ countries and jurisdictions, but not in the United States.
- GWLs can introduce negative affect when they remind smokers of the health consequences of smoking.

What important gaps in knowledge exist on this topic?

- There has been no systematic examination of the range of affect on current smokers produced by cigarette packaging with and without tobacco industry branding and with and without GWLs.
- An examination of real-world emotional responses to various components of packaging elements are needed to help inform how packaging designs may influence cognitions and behavior.

What this study adds?

- Handling current US branded cigarette packaging without GWLs was associated with moderate positive affect and feelings of trust and joy, an effect that was amplified when viewed immediately after exposure to packs without current branding but with GWLs.
- Handling blank packs (without GWLs and without current branding) was associated with lower positive affect than that associated with exposure to US branded packs without GWLs, regardless of prior exposure to packs without current branding but with GWLs.
- Handling 3 examples of packs without current branding but with GWLs induced a range of moderate to severe negative affect and provoked feelings of disgust, fear, anger, and sadness.
- Exposure to blank, branded and blank + GWLs packaging elicited immediate neutral, positive, and negative affect respectively. Different versions of the blank + GWL pack elicited different levels and types of negative affect.
- The immediate reactivity measure revealed the range of affect needed to explore the role of differentially valenced packaging on smokers’ cognitions and behavior in a real-world randomized trial.

## References

[1] World Health Organization. Plain packaging of tobacco products: evidence, design and implementation: World Health Organization, 2016.

[2] Moodie C, Angus K, Stead M, Plain tobacco packaging research: An update. 2013

[3] Pierce JP, Choi WS, Gilpin EA, Tobacco industry promotion of cigarettes and adolescent smoking. Jama 1998;279(7):511–15. 9480360 10.1001/jama.279.7.511

[4] Johnson SE, Coleman BN, Schmitt CL. It’s complicated: Examining smokers’ relationships with their cigarette brands. Psychology of Addictive Behaviors 2016;30(8):887. 27831717 10.1037/adb0000225 PMC5702884

[5] Zajonc RB, Markus H. Affective and cognitive factors in preferences. Journal of consumer research 1982;9(2):123–31.

[6] Tiffany ST, Drobes DJ. Imagery and smoking urges: The manipulation of affective content. Addictive behaviors 1990;15(6):531–39. 2075850 10.1016/0306-4603(90)90053-z

[7] Sherman DK, Kim HS. Affective perseverance: The resistance of affect to cognitive invalidation. Personality and Social Psychology Bulletin 2002;28(2):224–37.

[8] Berridge KC. Reward learning: reinforcement, incentives, and expectations. 2001

[9] Berridge KC, Robinson TE. Parsing reward. Trends in neurosciences 2003;26(9):507–13. 12948663 10.1016/S0166-2236(03)00233-9

[10] Countries with Pictorial Health Warning Labels, by Size [Available from: https://www.tobaccofreekids.org/assets/global/pdfs/en/GHWs_Size_List_July_2016.pdf accessed June 10, 2021.

[11] Scollo M, Lindorff K, Coomber K, Standardised packaging and new enlarged graphic health warnings for tobacco products in Australia—legislative requirements and implementation of the Tobacco Plain Packaging Act 2011 and the Competition and Consumer (Tobacco) Information Standard, 2011. Tobacco control 2015;24(Suppl 2):ii9–ii16. 28407605 10.1136/tobaccocontrol-2014-052073 PMC4401341

[12] Society CC. Cigarette package health warnings: international status report. Canadian Cancer Society 2018(sl)

[13] Shibuya K, Ciecierski C, Guindon E, WHO Framework Convention on Tobacco Control: development of an evidence based global public health treaty. Bmj 2003;327(7407):154–57. 12869461 10.1136/bmj.327.7407.154 PMC1126513

[14] Hall MG, Sheeran P, Noar SM, Negative affect, message reactance and perceived risk: how do pictorial cigarette pack warnings change quit intentions? Tobacco control 2017:tobaccocontrol-2017–053972. 10.1136/tobaccocontrol-2017-053972 PMC6004228 29248897

[15] Emery LF, Romer D, Sheerin KM, Affective and cognitive mediators of the impact of cigarette warning labels. Nicotine & Tobacco Research 2013;16(3):263–69. 23946325 10.1093/ntr/ntt124 PMC3920332

[16] Wang A-L, Lowen SB, Romer D, Emotional reaction facilitates the brain and behavioural impact of graphic cigarette warning labels in smokers. Tobacco control 2015;24(3):225–32. 25564288 10.1136/tobaccocontrol-2014-051993 PMC4495960

[17] Anshari D, Yong H-H, Borland R, Which type of tobacco product warning imagery is more effective and sustainable over time? A longitudinal assessment of smokers in Canada, Australia and Mexico. BMJ open 2018;8(7):e021983. 10.1136/bmjopen-2018-021983 PMC6089327 30007932

[18] Cho YJ, Thrasher JF, Swayampakala K, Does reactance against cigarette warning labels matter? Warning label responses and downstream smoking cessation amongst adult smokers in Australia, Canada, Mexico and the United States. PloS one 2016;11(7):e0159245. 27411100 10.1371/journal.pone.0159245 PMC4943644

[19] Cho YJ, Thrasher JF, Yong H-H, Path analysis of warning label effects on negative emotions and quit attempts: a longitudinal study of smokers in Australia, Canada, Mexico, and the US. Social Science & Medicine 2018;197:226–34. 29096946 10.1016/j.socscimed.2017.10.003 PMC5758420

[20] Mauss Iris B. & Michael D Robinson Measures of emotion: A review, Cognition and Emotion 2009;23:209. 19809584 10.1080/02699930802204677 PMC2756702

[21] Lee N, Broderick AJ, Chamberlain L. The application of physiological observation methods to emotion research. Qualitative Market Research: An International Journal 2007

[22] Russell JA, Barrett LF. Core affect, prototypical emotional episodes, and other things called emotion: dissecting the elephant. Journal of personality and social psychology 1999;76(5):805. 10353204 10.1037//0022-3514.76.5.805

[23] Noar SM, Rohde JA, Barker JO, Pictorial cigarette pack warnings increase some risk appraisals but not risk beliefs: a meta-analysis. Human communication research 2020;46(2–3):250–72. 32565612 10.1093/hcr/hqz016 PMC7291919

[24] Torre JB, Lieberman MD. Putting feelings into words: Affect labeling as implicit emotion regulation. Emotion Review 2018;10(2):116–24.

[25] Van Someren M, Barnard Y, Sandberg J. The think aloud method: a practical approach to modelling cognitive processes. London: AcademicPress 1994

[26] Koro-Ljungberg M, Douglas EP, Therriault D, Reconceptualizing and decentering think-aloud methodology in qualitative research. Qualitative Research 2013;13(6):735–53.

[27] Eccles DW, Arsal G. The think aloud method: what is it and how do I use it? Qualitative Research in Sport, Exercise and Health 2017;9(4):514–31.

[28] Hansen G Experience and emotion in empirical translation research with think-aloud and retrospection. Meta: journal des traducteurs/Meta: Translators’ Journal 2005;50(2):511–21.

[29] Hill RP, Mazis MB. Measuring emotional responses to advertising. ACR North American Advances 1986

[30] Renz SM, Carrington JM, Badger TA. Two strategies for qualitative content analysis: An intramethod approach to triangulation. Qualitative health research 2018;28(5):824–31. 29424274 10.1177/1049732317753586

[31] Francis DB, Hall MG, Noar SM, Systematic review of measures used in pictorial cigarette pack warning experiments. Nicotine & Tobacco Research 2017;19(10):1127–37. 28431080 10.1093/ntr/ntx082 PMC5896543

[32] Food U, Administration D. Tobacco products: Required warnings for cigarette packages and advertisements. Final rule Fed Regist 2020;85:15638–710. 21696017

[33] Pierce JP, Strong DR, Stone MD, Real-world exposure to graphic warning labels on cigarette packages in US smokers: The CASA randomized trial protocol. Contemporary Clinical Trials 2020;98:106152. 32966877 10.1016/j.cct.2020.106152 PMC7502239

[34] Watson D, Clark LA, Tellegen A. Development and validation of brief measures of positive and negative affect: the PANAS scales. Journal of personality and social psychology 1988;54(6):1063. 3397865 10.1037//0022-3514.54.6.1063

[35] Ziemer KS, Korkmaz G. Using text to predict psychological and physical health: A comparison of human raters and computerized text analysis. Computers in Human Behavior 2017;76:122–27.

[36] Zhang Y, Wildemuth BM. Qualitative analysis of content. Applications of social research methods to questions in information and library science 2009;308:319.

[37] Rinker T Package ‘sentimentr’. Retrieved 2017;8:31.

[38] Jockers ML. Syuzhet: Extract sentiment and plot arcs from text. Retrieved October 2015;21:2015.

[39] Schrauf RW, Sanchez J. The preponderance of negative emotion words in the emotion lexicon: A cross-generational and cross-linguistic study. Journal of multilingual and multicultural development 2004;25(2–3):266–84.

[40] Plutchik R A general psychoevolutionary theory of emotion. Theories of emotion: Elsevier 1980:3–33.

[41] Emotions evoked by common words and phrases: Using mechanical turk to create an emotion lexicon. Proceedings of the NAACL HLT 2010 workshop on computational approaches to analysis and generation of emotion in text; 2010.

[42] Plutchik R The nature of emotions: Human emotions have deep evolutionary roots, a fact that may explain their complexity and provide tools for clinical practice. American scientist 2001;89(4):344–50.

[43] Heatherton TF, Kozlowski LT, Frecker RC, The Fagerström test for nicotine dependence: a revision of the Fagerstrom Tolerance Questionnaire. British Journal of Addiction 1991;86(9):1119–27. 1932883 10.1111/j.1360-0443.1991.tb01879.x

[44] Simms LJ, Goldberg LR, Roberts JE, Computerized adaptive assessment of personality disorder: Introducing the CAT–PD project. Journal of personality assessment 2011;93(4):380–89. 22804677 10.1080/00223891.2011.577475 PMC3400119

[45] Moodie C, Mackintosh AM, Hastings G, Young adult smokers’ perceptions of plain packaging: a pilot naturalistic study. Tobacco control 2011;20(5):367–73. 21752795 10.1136/tc.2011.042911

[46] Moodie C, Stead M, Bauld L, Plain tobacco packaging: a systematic review. 2012 10.1136/bmj.f4786 23902856

[47] Hallgren KA. Computing inter-rater reliability for observational data: an overview and tutorial. Tutorials in quantitative methods for psychology 2012;8(1):23. 22833776 10.20982/tqmp.08.1.p023 PMC3402032

[48] McGraw KO, Wong SP. Forming inferences about some intraclass correlation coefficients. Psychological methods 1996;1(1):30.

[49] Porter MM, Niksiar P. Multidimensional mechanics: Performance mapping of natural biological systems using permutated radar charts. PloS one 2018;13(9):e0204309. 30265707 10.1371/journal.pone.0204309 PMC6161877

[50] Hammond D, Thrasher J, Reid JL, Perceived effectiveness of pictorial health warnings among Mexican youth and adults: a population-level intervention with potential to reduce tobacco-related inequities. Cancer Causes & Control 2012;23(1):57–67. 22362058 10.1007/s10552-012-9902-4 PMC4586036

[51] Thrasher JF, Carpenter MJ, Andrews JO, Cigarette warning label policy alternatives and smoking-related health disparities. American journal of preventive medicine 2012;43(6):590–600. 23159254 10.1016/j.amepre.2012.08.025 PMC3504356

[52] Hammond D, Reid JL, Driezen P, Are the same health warnings effective across different countries? An experimental study in seven countries. Nicotine and Tobacco Research 2019;21(7):887–95. 30452728 10.1093/ntr/nty248 PMC6588394

[53] Leas EC, Pierce JP, Dimofte CV, US adult smokers’ perceptions’ of Australia’s cigarette warning labels: variance by warning content and consistency across sociodemographic subsegments. Tobacco control 2017;26(4):485–86. 27343227 10.1136/tobaccocontrol-2016-053006 PMC5309205

[54] Hill D, Carroll T. Australia’s national tobacco campaign. Tobacco Control 2003;12(suppl 2):ii9–ii14. 12878768 10.1136/tc.12.suppl_2.ii9 PMC1766100

[55] Brennan E, Maloney EK, Ophir Y, Potential effectiveness of pictorial warning labels that feature the images and personal details of real people. Nicotine & Tobacco Research 2017;19(10):1138–48. 27932628 10.1093/ntr/ntw319 PMC5896434

[56] Hall MG, Sheeran P, Noar SM, Reactance to health warnings scale: development and validation. Annals of Behavioral Medicine 2016;50(5):736–50. 27333895 10.1007/s12160-016-9799-3 PMC5055422

[57] Strong DR, Pierce JP, Pulvers K, Effect of Graphic Warning Labels on Cigarette Packs on US Smokers’ Cognitions and Smoking Behavior After 3 Months: A Randomized Clinical Trial. JAMA Network Open 2021;4(8):e2121387–e87. 34347057 10.1001/jamanetworkopen.2021.21387 PMC8339936

[58] Borland R, Wilson N, Fong GT, Impact of graphic and text warnings on cigarette packs: findings from four countries over five years. Tobacco control 2009;18(5):358–64. 19561362 10.1136/tc.2008.028043 PMC4527864

[59] Thrasher JF, Swayampakala K, Borland R, Influences of self-efficacy, response efficacy, and reactance on responses to cigarette health warnings: a longitudinal study of adult smokers in Australia and Canada. Health communication 2016;31(12):1517–26. 27135826 10.1080/10410236.2015.1089456 PMC4972657

[60] Borland R, Wilson N, Fong GT, Impact of graphic and text warnings on cigarette packs: findings from four countries over five years. Tobacco control 2009;18(5):358–64. 19561362 10.1136/tc.2008.028043 PMC4527864

[61] Bolles RC. Reinforcement, expectancy, and learning. Psychological review 1972;79(5):394.

[62] Market Research Reports on tobacco plain packaging and graphic health warnings. Department of Health and Ageing, Austalia 2011

[63] Robinson TE, Berridge KC. The neural basis of drug craving: an incentive-sensitization theory of addiction. Brain research reviews 1993;18(3):247–91. 8401595 10.1016/0165-0173(93)90013-p

[64] Robinson TE, Flagel SB. Dissociating the predictive and incentive motivational properties of reward-related cues through the study of individual differences. Biological psychiatry 2009;65(10):869–73. 18930184 10.1016/j.biopsych.2008.09.006 PMC2737368

[65] Wakefield M, Coomber K, Zacher M, Australian adult smokers’ responses to plain packaging with larger graphic health warnings 1 year after implementation: results from a national cross-sectional tracking survey. Tobacco Control 2015;24(Suppl 2):ii17–ii25. 28407606 10.1136/tobaccocontrol-2014-052050 PMC4401339

[66] Miller CL, Ettridge KA, Wakefield MA. “You’re made to feel like a dirty filthy smoker when you’re not, cigar smoking is another thing all together.” Responses of Australian cigar and cigarillo smokers to plain packaging. Tobacco control 2015;24(Suppl 2):ii58–ii65. 28407613 10.1136/tobaccocontrol-2014-052049 PMC4401347

[67] White V, Williams T, Wakefield M. Has the introduction of plain packaging with larger graphic health warnings changed adolescents’ perceptions of cigarette packs and brands? Tobacco Control 2015;24(Suppl 2):ii42–ii49. 28407611 10.1136/tobaccocontrol-2014-052084 PMC4401345

[68] Pierce JP, Shi Y, McMenamin SB, Trends in lung cancer and cigarette smoking: California compared to the rest of the United States. Cancer prevention research 2019;12(1):3–12. 30305281 10.1158/1940-6207.CAPR-18-0341 PMC7389269

[69] Little RJ, Rubin DB. The analysis of social science data with missing values. Sociological Methods & Research 1989;18(2–3):292–326.

[70] Peters E, Evans AT, Hemmerich N, Emotion in the law and the lab: the case of graphic cigarette warnings. Tobacco regulatory science 2016;2(4):404–13. 29057296 10.18001/TRS.2.4.10 PMC5648023

