# Supplementary material for: The Role of Affective Reactivity Induced by Cigarette Packaging including Graphic Warning Labels: The CASA Study
Source: Tob Control. Author manuscript; Available in PMC 2024 May 1. (PMC8917242; doi:10.1136/tobaccocontrol-2021-056650)
Supplement: Supp1 [file NIHMS1736349-supplement-Supp1.pdf]

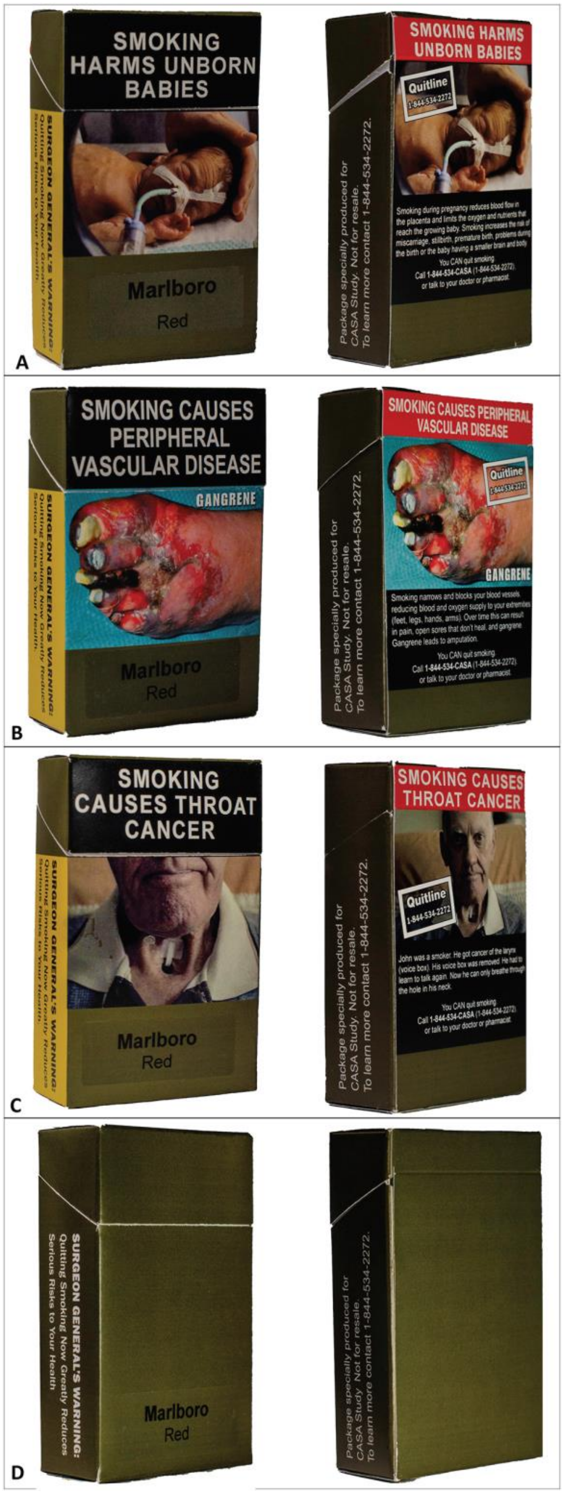

eFigure 1. Images of Manufactured Study Packs.

Note. (A) Neonatal Baby*, (B) Foot Gangrene*, (C) Throat Cancer*, (D) Blank Pack.
Reprinted from Contemporary Clinical Trials.[1]
* © Commonwealth of Australia

eReferences

1. Pierce JP, Strong DR, Stone MD, et al. Real-world exposure to graphic warning labels on cigarette packages in US smokers: The CASA randomized trial protocol. *Contemporary Clinical Trials.* 2020;98:106152.
