# Supplementary material for: The Role of Affective Reactivity Induced by Cigarette Packaging including Graphic Warning Labels: The CASA Study
Source: Tob Control. Author manuscript; Available in PMC 2024 May 1. (PMC8917242; doi:10.1136/tobaccocontrol-2021-056650)
Supplement: Supp2 [file NIHMS1736349-supplement-Supp2.pdf]

eTable 1. Examples of Natural Language Processing Estimates of the Average Speech Polarity that Smokers Uttered in Response to Viewing Study Packs

Quartile	Polarity	Example Text
Q1	-.461	Foot Gangrene Pack: *"Wow. That's gross. The foot, it catches my eye. It's disgusting looking. I've never seen anything like that. It's scary. Yeah. Just gross."*
	-.448	Neonatal Baby Pack: *"I think that would be very sad. I feel badly. Well, I suppose defensive because I smoked when I was pregnant, and my daughter was fine, I think. And hopefully I didn't cause any damage so maybe I'm feeling a little worried that I'm guilty of hurting her."*
	-.418	Throat Stoma Pack: *"It's scary. Painful. Devastating. No."*
Q2	-.183	Foot Gangrene Pack: *"So that's quite an unpleasant picture. Definitely wouldn't want to be in that situation. I imagine it has something to do with smoking, but I'm sure there's a whole lot more going on to get to this situation like that versus the other one that was, you know, more directly related."*
	-.169	Neonatal Baby Pack: *"Definitely the dying baby. Yeah, it makes me feel kind of guilty about smoking, but I mean I'm not pregnant, so it doesn't really affect me, I guess, but I don't know. It's kind of off-putting to know that my cigarettes are in here."*
	-.134	Throat Stoma Pack: *"Yeah. I mean, it's definitely something that, I'd rather quit before I get to that point. Yeah, it's a pretty unpleasant picture and, yeah, definitely doesn't look like a pleasant situation to be in."*
Q3	.000	Blank Pack: *"Plain. I don't really mind it so much. It's just a plain box. I actually almost kind of like it because it's plain, but it doesn't make me feel much. I don't really think of anything."*
	.000	Blank Pack: *"Nothing really on there, there's the warning label. Pretty much the warning label that catches your eye one you actually turn it over, assuming you actually turn it to the sides. Very plain."*
Q4	+.199	Own Pack: *"Kind of bright, pretty color, really can't see the Surgeon's General Warning, silver kind of blends in with the packaging. Yeah, cool looking pack."*
	+.516	Own Pack: *"These feel very familiar, very attractive. It's got the nice little gold bronze going on that I'm very used to. The descriptive words on the front, like smooth, rich, mellow, it's just attractive and very comfortable and familiar to me."*

Note. Using SentimentR, average paragraph level polarity scores were generated using the 'sentiment' function and sentence level polarity characteristics were generated using the 'highlight' function (positive = green; neutral = gray; negative = red). Polarity scores across the five pack conditions were pooled and divided into four even quartiles. Example statements and polarity scores are presented for each quartile, with each study pack condition appearing two times.

eTable 2. Sample Characteristics

Variable	Total (N=324)
Age[1]	39.3 (11.8)
Gender[2]	
Male	152 (47%)
Female	172 (53%)
Race/Ethnicity[2]	
White, Non-Hispanic	219 (68%)
Hispanic	35 (11%)
Other, Non-Hispanic	70 (22%)
American Indian/Alaska Native	4 (1.2%)
Asian	24 (7.4%)
Black or African American	12 (3.7%)
Native Hawaiian or Pacific Islander	5 (1.5%)
Other	19 (5.9%)
Decline to answer	6 (1.9%)
Education[2]	
College or Advanced Degree	134 (41%)
High School or less	40 (12%)
Some college	150 (46%)
Cigarettes per day[1]	11.6 (5.9)
Nicotine dependence (range: 0-10)[1]	3.8 (2.3)
Primary brand smoked[2]	
Marlboro	138 (43%)
American Spirit	58 (18%)
Camel	83 (26%)
Other	45 (14%)
Cigarette type	
Menthol	92 (28%)
Non-Menthol	232 (72%)
Brand loyalty[2]	
No	75 (23%)
Yes	249 (77%)
Brand appeal (range: 1-6)[1]	3.7 (1.2)
Health anxiety (range: 0-4)[1]	1.1 (0.9)

[1] Statistics presented: Mean (SD)
[2] Statistics presented: n (%)
